# Model-Based Design of Biochemical Microreactors

**DOI:** 10.3389/fbioe.2016.00013

**Published:** 2016-02-15

**Authors:** Tobias Elbinger, Markus Gahn, Maria Neuss-Radu, Falk M. Hante, Lars M. Voll, Günter Leugering, Peter Knabner

**Affiliations:** ^1^Chair of Applied Mathematics 1, Friedrich-Alexander-Universität Erlangen-Nürnberg, Erlangen, Germany; ^2^Chair of Applied Mathematics 2, Friedrich-Alexander-Universität Erlangen-Nürnberg, Erlangen, Germany; ^3^Chair of Biochemistry, Friedrich-Alexander-Universität Erlangen-Nürnberg, Erlangen, Germany

**Keywords:** biochemical microreactor, multienzymes complexes, spatio-temporal mathematical model, numerical simulation, PDE-constrained optimization, model-based design

## Abstract

Mathematical modeling of biochemical pathways is an important resource in Synthetic Biology, as the predictive power of simulating synthetic pathways represents an important step in the design of synthetic metabolons. In this paper, we are concerned with the mathematical modeling, simulation, and optimization of metabolic processes in biochemical microreactors able to carry out enzymatic reactions and to exchange metabolites with their surrounding medium. The results of the reported modeling approach are incorporated in the design of the first microreactor prototypes that are under construction. These microreactors consist of compartments separated by membranes carrying specific transporters for the input of substrates and export of products. Inside the compartments of the reactor multienzyme complexes assembled on nano-beads by peptide adapters are used to carry out metabolic reactions. The spatially resolved mathematical model describing the ongoing processes consists of a system of diffusion equations together with boundary and initial conditions. The boundary conditions model the exchange of metabolites with the neighboring compartments and the reactions at the surface of the nano-beads carrying the multienzyme complexes. Efficient and accurate approaches for numerical simulation of the mathematical model and for optimal design of the microreactor are developed. As a proof-of-concept scenario, a synthetic pathway for the conversion of sucrose to glucose-6-phosphate (G6P) was chosen. In this context, the mathematical model is employed to compute the spatio-temporal distributions of the metabolite concentrations, as well as application relevant quantities like the outflow rate of G6P. These computations are performed for different scenarios, where the number of beads as well as their loading capacity are varied. The computed metabolite distributions show spatial patterns, which differ for different experimental arrangements. Furthermore, the total output of G6P increases for scenarios where microcompartimentation of enzymes occurs. These results show that spatially resolved models are needed in the description of the conversion processes. Finally, the enzyme stoichiometry on the nano-beads is determined, which maximizes the production of glucose-6-phosphate.

## 1 Introduction

One of the greatest challenges in biology is to understand the fundamental principles on how evolution has selected networks to fulfill specific functional needs in the control of metabolism or transcription. Synthetic biology approaches may help to shed light on such principles by identifying modular functional units of a network and uncover how units can be linked together to yield new function (Bashor et al., 2010). There has already been early success in engineering simple regulatory circuits that recapitulate some of the behaviors of natural regulatory circuits, like, e.g., circuits that regulate gene expression oscillations, bistable switches, or circuits that perform combinatorial logic operations, see Boyle and Silver (2009) and Purnick and Weiss (2009) for reviews. However, synthetic biology approaches are not limited to this field. An other important area of synthetic biology is in the development of synthetic organelles, which host metabolic processes, and which are able to communicate with the outside environment *via* transport processes over semipermeable membranes, which can either be built from natural constituents or from synthetic polymers. Such robust bioreactors can be, e.g., applied in biotechnology or drug delivery for the production of bioactive ingredients (Roodbeen and van Hest, 2009; Marguet et al., 2013).

A fundamental principle in the development of bioreactors is compartmentation. Hereby, the strategy is to mimic cellular organization, see, e.g., Roodbeen and van Hest (2009). The presence of compartments (organelles) inside living cells allows for a better regulatory control over the biological processes that occur inside these compartments, e.g., pathways competing for intermediates can occur in spatially separated compartments and can be regulated differently. Furthermore, microcompartmentation by means of metabolic channeling prevents the loss of intermediates and minimizes competing cross-reactions. However, clearcut experimental evidence demonstrating the importance of metabolic channeling for metabolic flux *in vivo* is lacking. Mimicking the natural situation, nanoreactors can be built by encapsulating enzymes in vesicular compartments [as summarized in Peters et al. (2012)], and in addition microcompartmentation of enzymes can be achieved with the help of synthetic protein scaffolds (Chen and Silver, 2012). Microreactors based on microfluidic devices carrying out enzymatic processes, reviewed in Nomura et al. (2004) and Asanomi et al. (2011), have also been into the focus of research in the past years. Several methods used to immobilize enzymes are available, like, e.g., immobilization of enzymes on magnetic microparticles. An approach in the development of microreactors for biosynthesis is the creation of fluidic assay-based microreactors with membrane-bounded subcompartments, carrying out biochemical conversion, and allowing exchange of substrates and products between individual subcompartments.

**Figure 1:**
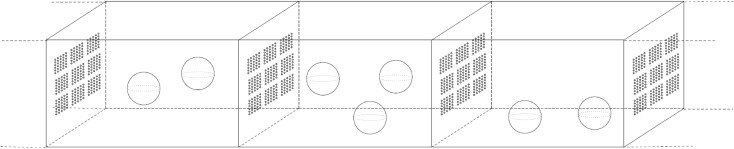
**A biochemical microreactor consisting of an array of compartments separated by membranes carrying specific transporters for input of substrates and export of products**. This microreactor is currently under construction and the modeling results reported here are being utilized to influence the conceptual design of the microreactor.

In our paper, we are focusing on the model-based design of biochemical microreactors able to carry out enzymatic reactions and to exchange metabolites between individual subcompartments. The bioreactor is built according to reported model predictions and is based on a microfluidic system consisting of chambers separated by porous walls, which are interspersed with biomembranes (Figure 1). The membranes carry specific transporters for input of substrates and export of products. Inside the chambers magnetic nano-beads are present that are positioned by an outer magnetic field.

Multienzyme complexes assembled on these nano-beads by peptide adapters are used to carry out metabolic reactions. The nano-beads allow a maximal enzyme concentration, which depends on the beads’ surface. Furthermore, it is possible to provide beads with a given enzyme stoichiometry. We exploit the microcompartmentation offered by the immobilized enzymes on the bead surface to address the question, if the spatial proximity of the individual enzymes and their stoichiometry has an impact on the productivity of the microreactor. In Figure 1, a microreactor consisting of an array of compartments is illustrated.

Our goal is to describe the spatio-temporal dynamics of metabolite concentrations involved in the biosynthetic conversions, and to optimize the microreactor, in order to increase the accumulation of the final product. To achieve this goal, we have developed a mathematical model describing the ongoing metabolic processes in spatial resolution. By applying a model with spatial resolution, we can assess, if the spatial arrangement of the individual enzymes and their stoichiometry has an impact on the productivity of the microreactor. Hence, our model will be able to predict, if metabolic channeling plays a role in the described *in vitro* assembly. Our model consists of a system of diffusion equations together with boundary and initial conditions. The boundary conditions model the exchange of metabolites with the neighboring chambers and the reactions at the surface of the nano-beads carrying the multienzyme complexes. For the numerical simulation of the mathematical model, efficient and accurate approaches are developed. These allow the computation of the spatio-temporal distribution of the metabolite concentrations inside the compartments and the flux of products through the export boundaries. Here, different computational scenarios can be considered including different numbers and loading capacities of nano-beads, different enzyme stoichiometries on the surface of the beads, as well as the distribution of enzymes both inside the fluid and on the beads’ surfaces. Comparing the metabolic flux through the system for those scenarios allow to test the hypothesis that microcompartmentation of enzymes increases the efficiency of the metabolic pathway. Based on the mathematical model and the simulation approach, the optimal design of the microreactor is performed.

As a proof-of-concept scenario, we choose a synthetic pathway for the conversion of sucrose to glucose-6-phosphate. This metabolic pathway is localized in one chamber of microreactor allowing the import of sucrose and the export of glucose-6-phosphate. Based on the mathematical model, several investigations are performed. First, the spatio-temporal distributions of the metabolite concentrations, as well as application relevant quantities like the production rate of the metabolites and outflow rate of G6P are computed. These computations are performed for different scenarios, where the number of beads as well as their loading capacity are varied. Furthermore, for the calculations, we consider two different hexokinases, namely HsHK2 and ScHK2, and we suppose sucrose and ATP to be present in surplus quantities. Finally, for the mentioned scenarios, we determine the stoichiometry of enzyme concentrations on the nano-beads which maximizes the production of glucose-6-phosphate.

## 2 Materials and Methods

### 2.1 The Mathematical Model

In this section, we set up a mathematical model describing the conversion of sucrose (S) to glucose-6-phosphate (G6P). This metabolic pathway is carried out in a microreactor consisting of a chamber separated from the surrounding medium by membranes carrying transport proteins for the input of sucrose and export of glucose-6-phosphate. Nano-beads loaded with multienzyme complexes are distributed inside the chamber. The enzymatic reactions constituting the metabolic pathway, as well as the corresponding enzymes and metabolites are listed in Table 1.

**Table 1:** **Metabolic reactions and the corresponding enzymes and metabolites**.

	Reactions	Enzymes
(0)	$S_{e}\;+\;{\text{H}}_{e}^{+}\;⇌\;S\;+\;{\text{H}}^{+}$	S-transporter
(1)	S → G + F	Invertase (inv)
(2)	G + ATP → G6P + ADP	Hexokinase (hk)
(3)	F + ATP → F6P + ADP	Hexokinase
(4)	F6P ⇌ G6P	Phosphoglucose isomerase (pgi)
(5)	G6P + Pi_e_ ⇌ G6P_e_ + Pi	G6P-transporter

*Metabolites: S, sucrose; H^+^, protons; G, glucose; F, fructose; G6P, glucose-6-phosphate; F6P, fructose-6-phosphate; Pi, inorganic phosphate*.

*The index *e* identifies metabolite located outside the chamber*.

**Figure 2:**
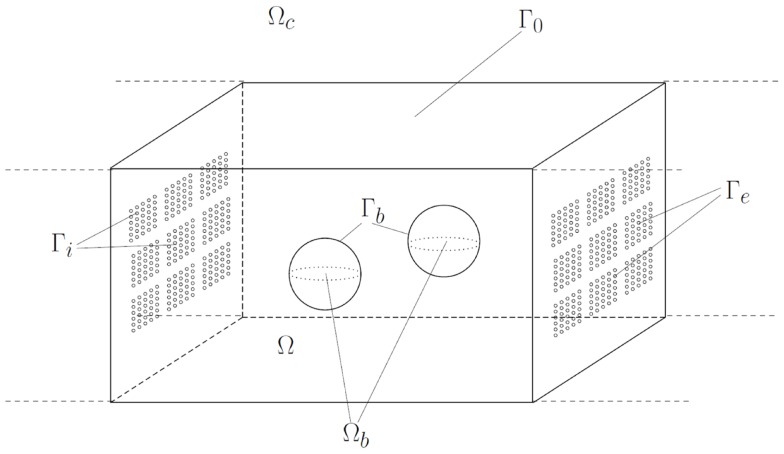
**Geometric representation of the modeled microreactor: reaction chamber Ω*_c_* with boundary ∂Ω*_c_* consisting of import boundary Γ*_i_*, export boundary Γ*_e_*, and impermeable boundary Γ_0_, and ensemble of nano-beads Ω*_b_* with reactive boundary Γ*_b_***.

**Figure 3:**
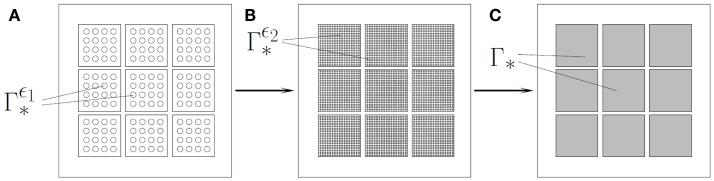
For pores of size ε > 0, the boundary part $\Gamma_{*}^{\epsilon }$, with * ∈ {*i,e*} contains the union of all pores in the inflow, respectively, outflow boundary. When performing the homogenization approach, we suppose that the number of the pores gets larger, while the size of the pores gets smaller, in such a way that the ratio between the surface occupied by pores and the surface of the fenestration remains constant. The value of this constant is the permeability θ*, * ∈ {*i,e*}, in the boundary conditions [equations (1c)–(1d)]. **(A)**. Inflow/outflow boundary for pores with size ε_1_. **(B)**. Inflow/outflow boundary for pores with size ε_2_ < ε_1_. **(C)**. *Via* homogenization, i.e., ε → 0, we obtain a homogenized model formulated on inflow/outflow boundaries without complex pore structure.

The layout of the microreactor is given in Figure 2. We denote the reactor chamber by Ω*_c_* ⊂ ℛ*^n^* (*n* = 2 or *n* = 3). The space inside the chamber occupied by nano-beads, which are balls with diameter *d*, is denoted by Ω*_b_*, whereas the remainder, representing the domain occupied by the bulk solution, is denoted by $\Omega :=\Omega_{c}\setminus \bar{\Omega_{b}}$. We assume that Ω*_b_* is strictly included in Ω*_c_*, this means that the beads do not touch the walls of the chamber. The number of beads in the reactor is denoted by *n_b_*. The boundary ∂Ω of the domain Ω is decomposed into ∂Ω*_c_*, the boundary of the chamber, and Γ*_b_*: = ∂Ω*_b_*, the reactive surface of the nano-beads. Furthermore, the boundary ∂Ω*_c_* of the chamber consists of Γ*_i_*, Γ*_e_*, and Γ_0_, i.e.,

$$\partial \Omega_{c}=\bar{\Gamma_{i}}\cup \bar{\Gamma_{e}}\cup \bar{\Gamma_{0}},$$

and the three sets are pairwise disjoint. The sets Γ*_i_*, respectively, Γ*_e_* represent the boundary parts where metabolites are transported into, respectively, out of the chamber Ω*_c_*. These import/export boundaries have a complex geometric structure. They consist of fenestrations lined with lipid-membranes carrying transporters for the exchange of metabolites. On the boundary Γ*_i_* the sucrose/proton cotransporters are distributed, whereas Γ*_e_* contains the transporters for the exchange of glucose-6-phosphate and inorganic phosphate. In our model, the microscopic structure of the boundaries is taken into account in an averaged (homogenized) way, which makes the model amenable for numerical calculations. More precisely, we assign to each of the boundaries Γ*_i_*, Γ*_e_* an effective permeability for the transported metabolites denoted by *θ^i^*, respectively, *θ^e^* (*θ^i^*, *θ^e^* ∈ [0,1]). These permeabilities can be calculated by an averaging approach (homogenization), assuming that the pores of the transporters are very small compared to the dimension of the reactor chamber and occur in a large number, and that the transporters are uniformly distributed within the lipid-membrane. A sketch illustrating the idea behind the homogenization approach is given in Figure 3.

The spatio-temporal dynamics of the concentrations *y_j_*, *j* = 1, … , *m* of metabolites involved in the metabolic pathway is governed by a system of reaction–diffusion equations of the form:

(1a) $$\partial_{t}y_{j}\left(t,x\right)-D_{j}\Delta y_{j}\left(t,x\right)=R_{j}^{\Omega }\left(\;y\left(t,x\right),\lambda \right)\text{ for}\;\left(t,x\right)\in \left(0,T\right)\times \Omega ,$$

together with the boundary conditions

(1b) $$\begin{array}{ll} & -D_{j}\nabla y_{j}\left(t,x\right)\cdot \nu \left(x\right)=-R_{j}^{b}\left(\;y\left(t,x\right),\lambda \right)\;\text{ for}\;\left(t,x\right)\in \left(0,T\right)\times \Gamma_{b},\; \end{array}$$

(1c) $$\begin{array}{ll} & -D_{j}\nabla y_{j}\left(t,x\right)\cdot \nu \left(x\right)=-\theta^{e}R_{j}^{e}\left(\;y\left(t,x\right)\right)\;\;\;\text{for}\;\left(t,x\right)\in \left(0,T\right)\times \Gamma_{e},\; \end{array}$$

(1d) $$\begin{array}{ll} & -D_{j}\nabla y_{j}\left(t,x\right)\cdot \nu \left(x\right)=-\theta^{i}R_{j}^{i}\left(\;y\left(t,x\right)\right)\;\;\text{for}\;\left(t,x\right)\in \left(0,T\right)\times \Gamma_{i}, \end{array}$$

(1e) $$\begin{array}{ll} & -D_{j}\nabla y_{j}\left(t,x\right)\cdot \nu \left(x\right)=0\;\;\;\;\;\text{for}\;\left(t,x\right)\in \left(0,T\right)\times \Gamma_{0}, \end{array}$$

and the initial condition

(1f) $$y_{j}\left(0,x\right)=y_{j}^{0}\left(x\right)\;\;\text{for}\;x\in \Omega .$$

Here, *ν* denotes the outer unit normal on ∂Ω with respect to Ω. Equation (1a) describes diffusion with diffusivity *D_j_* and enzymatic reactions with kinetics $R_{j}^{\Omega }$ for the metabolite number *j*. In case that enzymes are localized on beads (and thus no reactions are carried out in the bulk domain), the terms $R_{j}^{\Omega }$ are equal to zero. The boundary conditions involve the quantity −*D_j_*▽*y_j_* ⋅ *ν* which describes the normal component of the diffusive flux of the j-th metabolite at the boundary of the domain Ω. Thus, condition (1e) models an impermeable boundary, where the normal flux is equal to zero. Conditions (1c)–(1d) model the flux of metabolites through the import and export boundary, respectively. This flux is proportional to the permeability of the boundary, and the kinetics of the corresponding transport protein. Finally, condition (1b) describes the normal flux of metabolites at the boundary of the beads, generated by enzymatic reactions at the beads’ surface. We emphasize that the reaction rates $R_{j}^{b}$ and $R_{j}^{\Omega }$ depend on an additional parameter $\lambda =\left(\lambda_{1},\ldots ,\lambda_{n_{e}}\right)\in {ℛ}^{n_{e}}$. This parameter describes the stoichiometry of enzymes on the beads and in the bulk, and thus for *i* = 1, … , *n_e_*, where *n_e_* denotes the number of enzyme species involved in the reactions, holds

(2) $$\lambda_{i}\geq 0\;\;\;\text{and}\;\;\;\sum_{i=1}^{n_{e}}{\text{λ}}_{i}=1.$$

(The case λ_i__*_  = 1 for some i*  ∈ {1, … *n_e_*} means that all binding sites on the bead surface are occupied by the enzyme i*, whereas the case $\lambda_{i}=\frac{1}{n_{e}}$ for all *i* ∈ {1, … *n_e_*} means that all enzymes occupy the same amount of binding sites).

The existence and uniqueness of positive solutions for the model (1) can be shown by arguments similar to those in Gahn et al. (under review)^1^. We also emphasize that the model (1), valid for one conversion chamber, can easily be extended to model a multicompartment microreactor. This is done by adding transmission conditions at the interfaces separating the compartments, which model the exchange of metabolites between neighboring compartments.

In this paper, we want to investigate the interplay between the metabolic processes at the beads and the export of the product glucose-6-phosphate. We assume that during the conversion processes sucrose and ATP concentrations can be regarded as constant, since these two metabolite species are present in excess. As a consequence, we drop reaction (0). The equation for ADP can be neglected, since ADP is just a product of irreversible reactions and therefore has no direct effect on the reaction rates in equation (1). Finally, we assume that the concentrations of G6P_e_ and Pi_e_ (glucose-6-phosphate and inorganic phosphate outside the chamber) are constant. This is motivated by the fact that G6P_e_ may be consumed in a following reaction, and Pi_e_ may be delivered at a desirable rate in the space outside the chamber. Hence, we can drop the equations for G6P_e_ and Pi_e_. The constant values of the concentrations mentioned above are given by the corresponding initial concentrations (Table 3). The scenario taking into account these assumptions shall be referred to as the sucrose excess scenario (SE-scenario).

The reactions relevant for the SE-scenario are reactions (1)–(5) (Table 1). These are in general multisubstrate enzymatic reactions. Their reaction mechanisms and the corresponding reaction kinetics are displayed in Table 2A. Note that in Table 2, the concentrations of metabolites are denoted with brackets (e.g., for sucrose, we use [S] instead of *y_S_*). This is chosen in order to keep the notation clear.

**Table 2:** **(A) Metabolic reactions relevant for the sucrose excess scenario and the corresponding reaction kinetics; reaction mechanisms: (1) irreversible Michaelis–Menten; (2), (3) irreversible bi–bi ordered; (4) reversible Michaelis–Menten; (5) bi–bi ping pong. See, e.g., Segel (1975) for an overview on reaction mechanisms for multisubstrate enzymatic reactions; (B) reaction terms in equation (1) corresponding to the concentrations *y_j_*, *j* = G, … , G6P, for the SE-scenario with enzymes distributed on beads, i.e., ${\text{R}}_{\text{j}}^{\Omega }=0$**.

(A)		
Reaction	Reaction rate	
(1)	$r_{\text{inv}}\left(S\right)\;=\;\frac{\lambda_{\text{inv}}v_{\text{max}}^{\text{inv}}\left[S\right]}{K_{mS}+\left[S\right]}$	
(2)	$r_{\text{hk}}^{\text{G}}\left(\text{G},\text{ATP}\right)=\frac{\lambda_{\text{hk}}v_{\text{max}}^{\text{hk}}\left[\text{G}\right]\left[\text{ATP}\right]}{K_{m\text{G}}K_{m\text{ATP}}+K_{m\text{ATP}}\left[\text{G}\right]+K_{m\text{G}}\left[\text{ATP}\right]+\left[\text{G}\right]\left[\text{ATP}\right]}$	
(3)	$r_{\text{hk}}^{\text{F}}\left(\text{F},\text{ATP}\right)=\frac{\lambda_{\text{hk}}v_{\text{max}}^{\text{hk}}\left[\text{F}\right]\left[\text{ATP}\right]}{K_{m\text{F}}K_{m\text{ATP}}+K_{m\text{ATP}}\left[\text{F}\right]+K_{m\text{F}}\left[\text{ATP}\right]+\left[\text{F}\right]\left[\text{ATP}\right]}$	
(4)	$r_{\text{pgi}}\left(\text{F6P},\text{ G6P}\right)=\frac{\lambda_{\text{pgi}}v_{\text{max}}^{\text{pgi},f}\left[\text{F6P}\right]}{K_{m\text{F}6\text{P}}+\left[\text{F}6\text{P}\right]}-\frac{\lambda_{\text{pgi}}v_{\text{max}}^{\text{pgi},b}\left[\text{G6P}\right]}{K_{m\text{G}6\text{P}}+\left[\text{G}6\text{P}\right]}$	
(5)	$\begin{array}{cc} & r_{\text{G6P}}\left(\text{G6P},\text{ P}{\text{i}}_{\text{e}},\text{ G6}{\text{P}}_{\text{e}},\text{ Pi}\right)=\\ & \;\frac{\frac{v_{\text{max}}^{\text{G}6\text{P},f}\;\left(\left[\text{G}6\text{P}\right]\left[{\text{Pi}}_{\text{e}}\right]-\frac{\left[{\text{G}6\text{P}}_{\text{e}}\right]\left[\text{Pi}\right]}{K_{\text{eq}}}\right)}{\left[\text{G}6\text{P}\right]\left[{\text{Pi}}_{\text{e}}\right]+K_{m{\text{Pi}}_{\text{e}}}\left[\text{G}6\text{P}\right]+K_{m{\text{G}6\text{P}}^{\text{T}}}\left[{\text{Pi}}_{\text{e}}\right]\left(1+\frac{\left[\text{Pi}\right]}{K_{i\text{Pi}}}\right)+\ldots }}{\ldots \;\frac{v_{\max}^{\text{G}6\text{P, }f}}{v_{\max}^{\text{G}6\text{P, }b}K_{\text{eq}}}\;\left(K_{m\text{Pi }}[{\text{G}6\text{P}}_{\text{e}}]\;\left(1+\frac{\left[\text{G6P}\right]}{K_{i\text{G6P}}}\right)+\left[\text{Pi}\right]\left(K_{m{\text{G}6\text{P}}_{\text{e}}}+[{\text{G}6\text{P}}_{\text{e}}]\;\right)\right)} \end{array}$	

**(B)**		

**Species *j***	${\text{R}}_{\text{j}}^{\text{b}}$	${\text{R}}_{\text{j}}^{\text{e}}$

G	$r_{\text{inv}}\left(S\right)-r_{\text{hk}}^{\text{G}}\left(\text{G},\text{ATP}\right)$	0
F	$r_{\text{inv}}\left(S\right)-r_{\text{hk}}^{\text{F}}\left(\text{F},\text{ATP}\right)$	0
F6P	$r_{\text{hk}}^{\text{F}}\left(\text{F},\text{ATP}\right)-r_{\text{pgi}}\left(\text{F6P},\text{G6P}\right)$	0
Pi	0	*r*_G6P_(G6P, Pi_e_, G6P_e_, Pi)
G6P	$r_{\text{hk}}^{\text{G}}\left(\text{G},\text{ATP}\right)+r_{\text{pgi}}\left(\text{F6P},\text{G6P}\right)$	–r_G6P_ (G6P, Pi_e_, G6P_e_, Pi)

The unknowns of the model in the SE-scenario are the following metabolite concentrations: *y_G_*, *y_F_*, *y*_F6P_, *y*_Pi_, *y*_G6P_. For each unknown, a reaction–diffusion equation of type (1a) complemented by boundary conditions of type (1b)–(1e), and initial conditions holds. The reaction terms occurring in the equations are denoted by $R_{\text{G}}^{\Omega },\ldots ,R_{\text{G}6\text{P}}^{\Omega }$, whereas the fluxes at the boundaries Γ*_b_*, Γ*_e_*, Γ*_i_* are given by reaction terms denoted by $R_{\text{G}}^{b},\ldots ,R_{\text{G}6\text{P}}^{b}$, $R_{\text{G}}^{e},\ldots ,R_{\text{G}6\text{P}}^{e}$, and $R_{\text{G}}^{i},\ldots ,R_{\text{G}6\text{P}}^{i}$. The precise form of these reaction terms, in case when enzymes are distributed on beads, i.e., $R_{j}^{\Omega }=0$, is given in Table 2B. We emphasize that, when the metabolite *j*, *j*  = G, … , G6P participates in different reactions, the reaction term $R_{j}^{b}$ is given as a sum of all relevant reaction kinetics. If reactions take place also in the bulk, the reaction terms $R_{j}^{\Omega }$, for *j*  = G, … , G6P, have the same structure as $R_{j}^{b}$ with potentially different parameters. Finally, we mention that in the SE-scenario the inflow boundary Γ*_i_* is impermeable, thus the reaction terms $R_{\text{G}}^{i},\ldots ,R_{\text{G}6\text{P}}^{i}$ are set to zero.

The values $v_{\text{max}}^{i}$ for an enzyme *i, i* ∈ {inv, hk, pgi} in Table 2, can be calculated by $v_{\text{max}}^{i}=k_{\text{cat}}^{i}{\left[E\right]}_{0}$, with a turnover number $k_{\text{cat}}^{i}$ given in Table 3, and the concentration [*E*]_0_ of occupied binding sites on a bead. The enzymatic activity for the enzyme *i,i* ∈ {inv, hk, pgi} on each bead is then given by $\lambda_{i}v_{\text{max}}^{i}$, where the vector *λ* = (*λ*_inv_, *λ*_hk_, *λ*_pgi_) describes the enzymes stoichiometry on the bead. For the simulations, we choose $\lambda =\lambda^{E}:=\left(\lambda_{\text{ inv}}^{E},\lambda_{\text{hk}}^{E},\lambda_{\text{ pgi}}^{E}\right)$, where *λ^E^* satisfies

(3) $$\begin{array}{l} \begin{array}{rl} & k_{\text{cat}}^{\text{inv}}\lambda_{\text{inv}}^{E}=k_{\text{ cat}}^{\text{hk}}\lambda_{\text{hk}}^{E}=k_{\text{ cat}}^{\text{pgi}}\lambda_{\text{pgi}}^{E},\\ & \lambda_{\text{inv}}^{E}+\lambda_{\text{hk}}^{E}+\lambda_{\text{pgi}}^{E}=1. \end{array} \end{array}$$

**Table 3:** **Parameter values for the SE-scenario, corresponding to two different hexokinases HsHK2 and ScHK2**.

Parameter	Value	Parameter	Value
*K*_mS_	0.9 $\frac{\mathrm{mol}}{m^{\text{2}}}$	$k_{\text{cat}}^{\text{pgi}}$	646.61*s*^−1^
*K*_mG_	0.052 $\frac{\mathrm{mol}}{m^{\text{2}}}$ (HsHK2), 0.12 $\frac{\mathrm{mol}}{m^{\text{2}}}$ (ScHK2)	$v_{\text{max}}^{\text{inv},\text{full}}$	6.57 $\cdot {\text{10}}^{-\text{5}}\frac{\mathrm{mol}}{\mathrm{sm}}$
*K_m_*_ATP_	0.5 $\frac{\mathrm{mol}}{m^{\text{2}}}$ (HsHK2), 0.1 $\frac{\mathrm{mol}}{m^{\text{2}}}$ (ScHK2)	$v_{\text{max}}^{\text{hk},\text{full}}$	10.62 $\cdot {\text{10}}^{-\text{7}}\frac{\mathrm{mol}}{\mathrm{sm}}\left(\text{HsHK2}\right)$, 2.03 $\cdot {\text{10}}^{-\text{8}}\frac{\mathrm{mol}}{\mathrm{sm}}\left(\text{ScHK2}\right)$
*K_m_*_F_	11.4 $\frac{\mathrm{mol}}{m^{\text{2}}}$ (HsHK2), 0.33 $\frac{\mathrm{mol}}{m^{\text{2}}}$ (ScHK2)	$v_{\text{max}}^{\text{pgi},\text{full}}$	12.57 $\cdot {\text{10}}^{-\text{6}}\frac{\mathrm{mol}}{\mathrm{sm}}$
*K_m_*_F6P_	0.19 $\frac{\mathrm{mol}}{m^{\text{2}}}$	$v_{\text{max}}^{\text{G6P},\text{f}}$	3.23 $\cdot {\text{10}}^{-\text{8}}\frac{\mathrm{mol}}{\mathrm{sm}}$
*K_m_*_G6P_	0.5 $\frac{\mathrm{mol}}{m^{\text{2}}}$	$v_{\text{max}}^{\text{G6P},\text{b}}$	3.23 $\cdot {\text{10}}^{-\text{8}}\frac{\mathrm{mol}}{\mathrm{sm}}$
$K_{m{\text{G6P}}^{\text{T}}}$	0.7 $\frac{\mathrm{mol}}{m^{\text{2}}}$	*λ^E^*	(0.0147, 0.91, 0.0753) (HsHK2)
$K_{m{\text{G6P}}_{\text{e}}}$	0.7 $\frac{\mathrm{mol}}{m^{\text{2}}}$	*λ^E^*	(0.0003, 0.9981, 0.0016) (ScHK2)
*K_m_*_Pi_	1.1 $\frac{\mathrm{mol}}{m^{\text{2}}}$	*θ^e^*	0.15
$K_{m{\text{Pi}}_{\text{e}}}$	1.1 $\frac{\mathrm{mol}}{m^{\text{2}}}$	[*G*6*P_e_*]	1$\frac{\mathrm{mol}}{m^{\text{2}}}$
*K_i_*_G6P_	0.9 $\frac{\mathrm{mol}}{m^{\text{2}}}$	[*PI*_e_]	10$\frac{\mathrm{mol}}{m^{\text{2}}}$
*K_i_*_Pi_	0.1 $\frac{\mathrm{mol}}{m^{\text{2}}}$	[*ATP*]	10$\frac{\mathrm{mol}}{m^{\text{2}}}$
*K*_eq_	0.5 $\frac{\mathrm{mol}}{m^{\text{2}}}$	[*S*]	50$\frac{\mathrm{mol}}{m^{\text{2}}}$
$k_{\text{cat}}^{\text{hk}}$	54.63*s*^−1^(HsHK2), 1.06*s*^−1^(ScHK2)	*d*	10^−5^*m*
$k_{\text{cat}}^{\text{inv}}$	3379.60*s*^−1^	[*E*]_full_	1.94 $\cdot {\text{10}}^{-\text{8}}\frac{\mathrm{mol}}{m}$

*These parameters correspond to the 2-dimensional case and their units of measurements were adapted to this case. Approaches for the experimental determination of the *k_cat_*-values can be found in Gao and Leary (2003), Gloyn et al. (2005), Lin et al. (2009), Lafraya et al. (2011), and Somarowthu et al. (2011). We use the values below to calculate the *ν*_max_-values. The initial values $y_{j}^{0}$ in equation (6) for the species *j* = G, F, F6P, G6P, Pi are equal to zero. For the diffusion–coefficients, *D_j_* belonging to these species we use the value $D_{j}=\text{5.5}\cdot {\text{10}}^{-\text{10}}\frac{m^{\text{2}}}{s}$*.

This enzyme stoichiometry leads to equal enzymatic activity for all enzyme species. The values of *λ^E^* are given in Table 3. Please note that in Section 2.3 optimal values for the parameter *λ* are computed.

### 2.2 Numerical Methods

We use a finite element method in order to find an approximation to the solution of equation (1) on a fixed time interval [0, *T*] and with a given initial state.

For the numerical accuracy and the computational complexity of an implementation, also in view of the optimization, it is crucial to choose a suitable discretization. For our problem, we use lowest order Raviart–Thomas elements (Raviart and Thomas, 1977). On a first glance, linear finite elements seem superior to Raviart–Thomas elements due to a lower number of unknowns and a higher order of convergence. However, for our problem, it turns out that the use of linear finite elements leads to solutions with negative concentrations when the triangulation𝒯*_h_* is not extremely fine. More precisely, for *n_b_* = 1 we investigated two scenarios, and compared the number of unknowns that are needed for each discretization, to obtain realistic results. For Ω*_c_* = (0.50 μm)^2^, we need 3200 degrees of freedom per species in order to obtain realistic results for linear finite elements, whereas Raviart-Thomas elements only need 993 degrees of freedom. For Ω*_c_* = (0.300 μm)^2^ we need 122,544 degrees of freedom per species in order to obtain realistic results for linear finite elements, whereas Raviart–Thomas elements only need 38,256 degrees of freedom. The computational complexity for Raviart–Thomas elements can be further reduced by hybridization, in this case only 607 degrees of freedom for the smaller domain and 23,016 degrees of freedom for the larger domain are needed.

The resulting space-discrete system is stiff, therefore, we use the implicit Euler method for the time integration. The resulting finite dimensional non-linear problems are solved with Newton’s method. Whenever the non-linear solver can not find a non-negative solution, the time step is rejected and a smaller time step size is used (until the non-linear solver can find a non-negative solution or the time step size is less than a predefined minimal time step size). We emphasize that decreasing the time step size was not necessary on the meshes used for spatial discretization (see Supplementary Material) with Raviart–Thomas elements and a time step size of 0.25 s.

In order to decrease the computational complexity of problem (1), arising from the number of species, we use the scheme presented in Kräutle and Knabner (2005) with minor modifications: instead of finding the basis of the image of the stoichiometric matrix *S* on the right hand side of the partial differential equation, we find the basis of the image of the matrix (*S_b_*|*S_e_*), where *S_x_* refers to the stoichiometric matrix of the reactions on Γ*_x_*, *x* ∈ {*b,e*}. The decoupled problem consists of 6 coupled species, whereas the original problem consists of 11 coupled species. In the SE-scenario, the decoupling scheme does not decrease the computational complexity, since the resulting stoichiometric matrix has full rank.

The numerical scheme has been implemented using the software package M++ that is based on the data structure in Wieners (2005). The algorithm uses MPI parallelization and can therefore use an arbitrary number of processors for computation. This is particularly useful for problems involving many species.

### 2.3 Optimization Methods

Besides setting up a mathematical model for the microreactor to describe the temporal and spatial distribution of enzymes and reactants, our goal also was to determine optimal parameters for enhanced performance of the microreactor based on this model. We demonstrate here how to succeed in a systematical way using derivative-based non-linear optimization techniques. Exemplary, we focus on how to determine the real parameters *λ* modeling the stoichiometry of the enzymes loaded on the beads entering in the non-linear terms in equation (1). We then discuss extensions of such methods to other parameters such as the discrete number of beads *n_b_*, the combinatorial problem of which specific enzymes should be used for the metabolic pathway and continuous geometry parameters such as the bead diameters or locations, the total activity of the transporter proteins, the shape and size of the chamber itself, etc.

The criterion for the performance of the reactor will be the total outflow of a desired product, say $y_{j_{*}}$(*λ*) on Γ*_e_*, over a fixed production time horizon [0,*T*],

(4) $$\begin{array}{llll} J\left(\;y\right) & =\int_{0}^{T}\;\int_{\Gamma_{e}}\;-D_{j_{*}}\nabla y_{j_{*}}\left(t,x\right)\cdot \nu \left(x\right)\;d\sigma \left(x\right)\;\mathrm{dt}\; & & \;\\ & =\int_{0}^{T}\;\int_{\Gamma_{e}}\;-\theta^{e}R_{j_{*}}^{e}\left(\;y\left(t,x\right)\right)\;d\sigma \left(x\right)\;\mathrm{dt}, \end{array}$$

where we have used equation (1c) for $j=j_{*}$ in equation (4). For our sucrolytic chamber, we have $j_{*}=j_{\text{G6P}}$. By optimal parameters, we mean that any feasible choice close to the optimal one does not lead to a higher total outflow predicted by the model.

While derivative-based non-linear optimization techniques are conceptually well-known, we emphasize that due to the large-scale, non-linear system of equations to be solved for each direct simulation of the model, the main challenge is an efficient computation of the derivative of the reduced cost function

(5) Ĵ(λ)=( y(λ)),

where *y*(*λ*) denotes the numerical solution of the system [equations (1a)–(1f)] as a function of the parameter *λ*. We will exploit that the dimension of the parameter space *n_p_* is typically small compared to the dimension obtained from discretization of the infinite-dimensional state space for *y* in the model and will therefore follow a sensitivity-based approach to compute the derivative [see, e.g., Hinze et al. (2009)]. Letting *e_i_*, *i* = 1, … , *n_p_*, being a bases of the parameter space this means that we can compute the derivative Ĵ′(λ) as

(6) $$Ĵ\prime \left(\lambda \right)=\sum_{i=1}^{l}\;J_{y}\left(\;y\left(\lambda \right)\right)\;\delta_{e_{i}}y,$$

where the sensitivity $\delta_{e_{i}}y=y\prime \left(\lambda \right)e_{i}$ is given by the solution (${\tilde{y}}_{1},\ldots ,{\tilde{y}}_{m}$) of the following linearized problem

(7a) $$\begin{array}{llll} & \partial_{t}{\tilde{y}}_{j}\left(t,x\right)-D_{j}\Delta {\tilde{y}}_{j}\left(t,x\right)=\partial_{y}R_{j}^{\Omega }\left(\;y\left(t,x\right),\lambda \right){\tilde{y}}_{j}\; & & \;\\ & \;-\partial_{\lambda }R_{j}^{\Omega }\left(\;y\left(t,x\right),\lambda \right)e_{i}\;\;\text{for}\;\left(t,x\right)\in \left(0,T\right)\times \Omega , \end{array}$$

together with the boundary conditions

(7b) $$\begin{array}{llll} & -D_{j}\nabla {\tilde{y}}_{j}\left(t,x\right)\cdot \nu \left(x\right)=-\partial_{y}R_{j}^{b}\left(\;y\left(t,x\right),\lambda \right){\tilde{y}}_{j}\; & & \;\\ & \;+\partial_{\lambda }R_{j}^{b}\left(\;y\left(t,x\right),\lambda \right)e_{i}\;\;\text{for}\;\left(t,x\right)\in \left(0,T\right)\times \Gamma_{b}, \end{array}$$

(7c) $$\begin{array}{llll} & -D_{j}\nabla {\tilde{y}}_{j}\left(t,x\right)\cdot \nu \left(x\right)=\; & & \;\\ & \;-\theta^{e}\left(R_{j}^{e}\right)\prime \left(\;y\left(t,x\right)\right){\tilde{y}}_{j}\;\;\text{for}\;\left(t,x\right)\in \left(0,T\right)\times \Gamma_{e}, \end{array}$$

(7d) $$\begin{array}{llll} & -D_{j}\nabla {\tilde{y}}_{j}\left(t,x\right)\cdot \nu \left(x\right)=\; & & \;\\ & \;-\theta^{i}\left(R_{j}^{i}\right)\prime \left(\;y\left(t,x\right)\right){\tilde{y}}_{j}\;\;\text{for}\;\left(t,x\right)\in \left(0,T\right)\times \Gamma_{i}, \end{array}$$

(7e) $$\begin{array}{ll} & -D_{j}\nabla {\tilde{y}}_{j}\left(t,x\right)\cdot \nu \left(x\right)=0\;\;\;\text{for}\;\left(t,x\right)\in \left(0,T\right)\times \Gamma_{0}, \end{array}$$

and the initial condition

(7f) $${\tilde{y}}_{j}\left(0,x\right)=0\;\;\text{for}\;x\in \Omega ,$$

where ∂*_y_* and ∂*_λ_* denote partial derivatives and ()′ [e.g., ($R_{j}^{i}$)′] denotes total derivatives of the corresponding kinetic functions, respectively, and where *y* is the solution of equations (1a)–(1f). The solution of this linearized problem can be computed simultaneously with the direct simulation using the same discretization method as described in Section 2.2. Compared to the evaluation of the objective function (5), the additional computational effort to obtain the derivative is solving *n_p_* linear equation systems in each time step. Instead of using the linearized problem (7), we can also obtain a derivative from an adjoint problem, see again, e.g., Hinze et al. (2009). The latter approach is more efficient when *n_p_* is large.

With the derivative at hand, we can then solve the reduced optimization problem subject to further parameter constraints for instance with primal-dual interior-point algorithms using a quasi-Newton approximation of the Hessian. These methods are known to have both good theoretical and practical behavior, see, e.g., Forsgren et al. (2002). Given some initial parameter *λ*^(0)^, they compute a sequence of parameters *λ*^(^*^k^*^)^, *k* = 1, 2, 3, …, converging to an optimal *λ* until for some k* first order conditions or stationarity in the objective function are satisfied within a tolerance tol*_X_* or tol_fun_, respectively. We set λ* = λ^(^^k^^*)^, J* = *J*(*y*(λ*)), and *J*^0^ = *J*(*y*(*λ*^(0)^)).

In order to determine an optimal stoichiometry *λ* for our sucrolytic chamber, we have the three parameters *λ*_inv_, *λ*_hk_, and *λ*_pgi_, but we may eliminate for example *λ*_pgi_ by the condition (2). This yields *n_p_* = 2, together with a linear inequality constraint *λ*_inv_ + *λ*_hk_ ≤ 1 and box constraints *λ*_inv_, *λ*_hk_ ∈ [0,1]. For our numerical results in Section 3.2 for the SE-scenario, we have used the interior-point algorithm implemented in the Optimization Toolbox in MATLAB (2014) with a BFGS quasi-Newton approximation of the hessian, where equations (5) and (6) are computed using the methods described in Section 2.2. Again we use the software package M++ for our implementation. As initial parameter *λ*^(0)^, we either use the stoichiometry from equal activity *λ^E^* given by equation (3) or the uniform stoichiometry $\lambda^{\left(0\right)}=\lambda^{U}=\left(\frac{1}{3},\frac{1}{3},\frac{1}{3}\right)$ depending on which has a larger value *J*^(0)^. Furthermore, we use tol*_X_* = 1.0*e*–06 and tol_fun_ = 1.0*e*–04 for all our optimization runs.

The derivative computation and hence the above method can directly be adapted to other real parameters entering the kinetic functions such as, e.g., the permeability *θ*. Combinatorial problems from (additional) discrete parameters such as the number of beads can be solved using enumeration or, approximately, by derivative-based optimization techniques based on relaxation methods, even for time dependent parameters as in Hante and Sager (2013). Continuously varying geometry parameters such as bead diameters or shape and size of the chamber Ω*_c_* can be handled similarly by shape derivatives as in Leugering et al. (2011). For the SE-scenario, we enumerate optimal λ* for various combinations of number of beads *n_b_*, different choices of hexokinases in the sucrolytic pathway and different time horizons *T*.

## 3 Results

We apply our simulation and optimization methods to the situation of the microreactor in the SE-scenario for realistic parameters. Hereby, we consider two-dimensional quadratic reaction chambers Ω*_c_* = (0, *L*)^2^ with *L* = 500 and *L* = 3000 μm, and different numbers of uniformly distributed beads, each of diameter of 10 μm. The outflow boundary is Γ*_e_* = {*L*} × (0, *L*). The rest of the chambers’ boundary is impermeable. The parameters of the mathematical model are given in Table 3. The final time *T* varies between 0.5 and 4 h. This is appropriate, due to the fact that the outflow-membrane Γ*_e_* is in general unstable and bursts after several hours. We stress that, due to the need of small time step sizes and the ratio of bead and chamber size, larger time horizons and chamber sizes lead to a higher computational complexity.

For our numerical investigations, we consider two types of hexokinase, namely HsHK2 and ScHK2. We also have two stoichiometry values which play a role in our calculations: the uniform stoichiometry $\lambda^{U}=\left(\frac{1}{3},\frac{1}{3},\frac{1}{3}\right)$, and the equal activity stoichiometry *λ^E^* described in equation (3). More precisely, *λ^E^* is used for the numerical simulations, whereas either of the two is used as initial parameter in the optimization, depending on which leads to a larger value of the total glucose-6-phosphate outflow.

### 3.1 Simulation Results

We consider a quadratic chamber Ω*_c_* with a length of 3000 μm, a fixed run time of *T* = 3 h, a fixed enzyme stoichiometry *λ* = *λ^E^* and a number of beads *n_b_* ∈ {0^2^, 1^2^, 2^2^, …, 6^2^, 12^2^, 18^2^, …, 60^2^}, where *n_b_* = 0 refers to a scenario with enzymes distributed in the bulk solution.

The meshes used for spatial discretization consist of between 15,190 triangles and 22,941 edges for 1 bead, and 93,184 triangles and 151,592 edges for 2916 beads. Detailed information about the meshes is available in the Supplementary Material. We used a time step size of 0.25 s. All calculations have been repeated for *T* = 1.5 h on uniform refined meshes and half of the time step size. No significant differences between the results on the coarse and on the fine meshes were observed.

**Figure 4:**
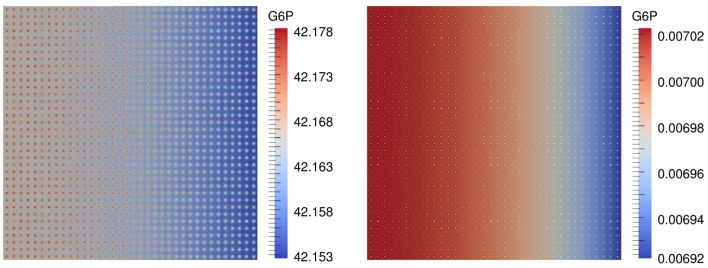
**G6P concentration in $\frac{\text{mol}}{m^{\text{2}}}$ in a scenario with *n_b_* = 1296 beads and the hexokinase HsHK2, for a completely loaded beads (left) and partially loaded beads, where the total number of enzymes distributed to the beads corresponds to that on one completely loaded bead (right), after 3 h**.

First results are concerned with the numerical computation of the spatial and temporal distribution of the metabolites. Figure 4 shows the distribution of G6P for a scenario with 1296 beads and the hexokinase HsHK2 after 3 h for completely and partially loaded beads.

Next results are concerned with the computation of quantities, which are particularly relevant for our microreactor, namely the export of G6P at time *t* ∈ [0,*T*], i.e., the outflow rate at time *t* given by

(8) $$\int_{\Gamma_{e}}\;-D_{\text{G}6\text{P}}\nabla y_{\text{G}6\text{P}}\left(t,x\right)\cdot \nu \left(x\right)d\sigma \left(x\right),$$

and the production rate for the metabolites at the beads at time *t* given by

(9) $$\int_{\Gamma_{b}}\;D_{j}\nabla y_{j}\left(t,x\right)\cdot \nu \left(x\right)d\sigma \left(x\right),$$

for *j* = G, … , G6P. In computing these quantities, we consider the following arrangements:

(S.1) Increase the number of beads *n_b_*, where beads are completely loaded.
(S.2) The total number of enzymes in the domain is fixed (and given by the number of binding sites on one completely loaded bead) and enzymes are distributed uniformly on *n_b_* beads or in bulk. The latter case, we characterize by *n_b_* = 0.

Both arrangements are simulated for the two hexokinases HsHK2 and ScHK2, and the beads are distributed periodically in the bulk, like, e.g., in Figure 4. For the arrangement (S.2), we also consider the case without beads where all enzymes are distributed in the bulk. Then the reaction terms $R_{j}^{\Omega }$ in equation (1a) are equal to $R_{j}^{b}$ from Table 2. In (S.1), the maximal velocity $v_{\max}^{i}=k_{\text{cat}}^{i}{\left[E\right]}_{0}$ corresponding to enzyme *i, i* ∈ {inv, hk, pgi} is calculated with [*E*]_0_ = [*E*]_full_ where [*E*]_full_ is the concentration of binding sites on one bead. These values are denoted by $v_{\max}^{i,\text{full}}$, and are given in Table 3. In (S.2), we fix the total concentration of enzyme to be given by [*E*]_full_. However, this concentration is now distributed on *n_b_* beads. Thus, the values for $v_{\max}^{i},i\in \;\left\{\text{inv},\text{hk},\text{pgi}\right\}$ are now obtained by dividing the values used in (S.1) by the number of beads *n_b_*.

**Figure 5:**
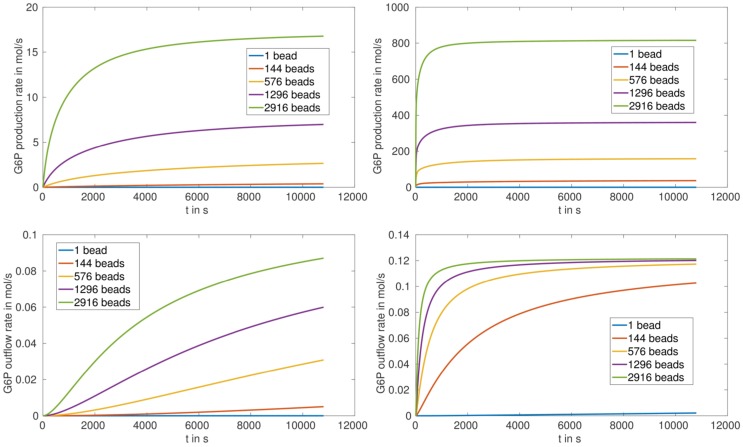
**G6P production rate (top) and outflow rate (bottom) in $\frac{\text{mol}}{s}$ for arrangement (S.1) with *n_b_* ∈ {1, 144, 576, 1296, 2916} and the enzymes ScHK2 (left) and HsHK2 (right)**.

**Figure 6:**
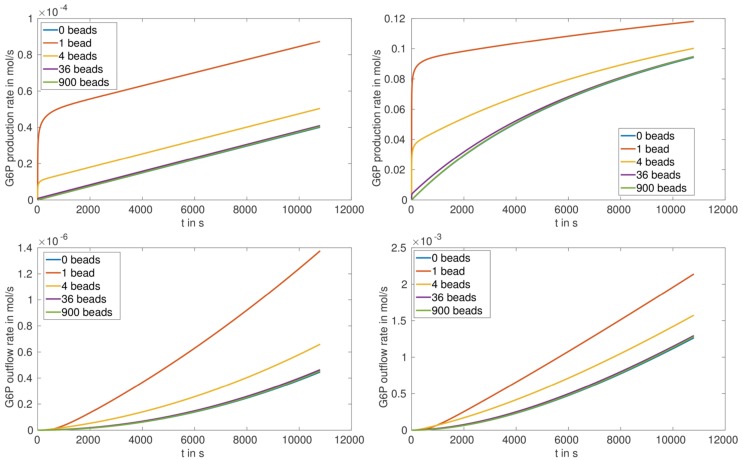
**G6P production rate (top) and outflow rate (bottom) in $\frac{\text{mol}}{s}$ for the arrangement (S.2) *n_b_* ∈ {0, 1, 4, 36, 900} and the enzymes ScHK2 (left) and HsHK2 (right)**.

The production and outflow rate for G6P are plotted in Figure 5 for (S.1) and Figure 6 for (S.2). For fully loaded beads the production rate increases with increasing number of beads, and reaches saturation after approximately 1000 s in the case of HsHK2 and after approximately 10,800 s in the case of ScHK2 (Figure 5, upper pictures). Concerning the outflow rate, in case of HsHK2 (Figure 5 right bottom) an upper bound is asymptotically reached, the value of which seams to be the same for all *n_b_* ≥ 576 (simulations with longer runtime suggest that the value seams to be the same for *n_b_* ≥ 16).

In case of partially loaded beads corresponding to arrangement (S.2), the production rate of G6P at the beads up to a time of 3 h first decreases with increasing number of beads and then stays nearly constant (Table 4; Figure 6, upper figures). Please note, that for the arrangement (S.2) the outflow rates are not reaching saturation, which implies that the transporters activity are not limiting the outflow for the chosen enzyme concentration [*E*]_full_. We also remark that for the chosen stoichiometry *λ^E^*, in case of arrangement (S.2), the production rate of G6P for HsHK2 is by a factor 10^3^ to 10^4^ larger than for ScHK2, i.e., in (S.2) the selected hexokinase has a greater influence on the conversion process as in (S.1).

**Table 4:** **Production and outflow rates at *T* = 3*h* for scenario (S.2) with the hexokinase HsHK2 and *n_b_* ∈ {1^2^, 2^2^, 6^2^, 18^2^, 30^2^, 42^2^, 54^2^, 0}, and times until half of these rates are reached**.

	*n_b_*	1	4	36	324	900	1764	2916	0
Rates after 3 h (in $\frac{\mathrm{mol}}{s}$)	Production	0.12	0.10	0.095	0.094	0.095	0.095	0.095	0.094
	Outflow	0.0021	0.0016	0.0013	0.0013	0.0013	0.0013	0.0013	0.0013
Time until half of the rate above is reached (in seconds)	Production	2	1494	3460	3603	3606	3600	3590	3618
	Outflow	5959	6457	7025	7090	7092	7090	7086	7096

In order to quantify how fast production and outflow rate for various number of beads increase in time in the scenario (S.2) with the hexokinase HsHK2, in Table 4, we give the values of these rates at *T* = 3 h, and the times the reactor needs to reach half of these values.

### 3.2 Optimization Results

We aim at finding the optimal stoichiometry λ* for the SE-scenario. Since our focus is on computing a variety of different scenarios with a reasonable computational effort, we have chosen the smaller chamber Ω*_c_* = (0.500 μm)^2^ for these experiments. The meshes used for the spatial discretization of this chamber consist of between 820 triangles and 1270 edges for 1 bead, and 2394 triangles and 3883 edges for 64 beads. Further information about the meshes is available in the Supplementary Material.

For our study, we consider the following arrangements:

(O.1) For a fixed runtime of *T* = 1 h, we increase the number of completely loaded beads in the chamber with *n_b_* ∈ {1, 4, 9, 16, 25, 36, 49, 64}.
(O.2) For a sucrolytic chamber with one completely loaded bead, we optimize the G6P outflow for different runtimes *T* ∈ {0.5, 1, 1.5, 2, … 4} hours.

We study these arrangements for the hexokinases ScHK2 and HsHK2.

For the enzyme ScHK2, we find in case of arrangement (O.1) that the optimal stoichiometry for a runtime of 1 h is between 3 and 12% for invertase, between 82 and 94% for hexokinase and between 3 and 6% for phosphoglucose isomerase. With an increasing number of beads, the proportion of ScHK2 increases and the proportions of invertase and phosphoglucose isomerase decrease. The exact results are listed in Table 5, where for each number of beads *n_b_*, the optimal stoichiometry λ* as well as the corresponding total outflow J* are displayed. From the arrangement (O.2), we find that the optimal stoichiometry for one bead is between 8 and 13% for invertase, between 80 and 87% for hexokinase and between 5 and 6% for phosphoglucose isomerase, again with an increase in the proportion of binding sites loaded with ScHK2 in expense of proportion for ScINV, but with an almost constant proportion of binding sites loaded with ScPGI when the runtime is successively increased. The exact results are listed in Table 6, where for each run time *T*, the optimal stoichiometry λ* as well as the corresponding total outflow J* are displayed.

**Table 5:** Optimal parameters λ* for the SE-scenario with ScHK2 (top) and HsHK2 (bottom), for a fixed runtime *T* = 3600, and different numbers *n_b_* of beads.

Enzyme	*n_b_*	****λ****^(0)^	*J*^0^	k*	$\lambda {\;}_{\text{inv}}^{*}$	$\lambda {\;}_{\text{hk}}^{*}$	$\lambda {\;}_{\text{pgi}}^{*}$	J*	$\frac{J^{*}-J^{\text{0}}}{J^{\text{0}}}$
ScHK2	1	*λ^U^*	0.228066	16	0.120404	0.815447	0.064149	0.446978	0.9599
ScHK2	4	*λ^U^*	0.968913	11	0.084901	0.860564	0.054536	2.090510	1.1576
ScHK2	9	*λ^U^*	2.144680	19	0.063959	0.895103	0.040938	4.686620	1.1852
ScHK2	16	*λ^U^*	3.652360	14	0.052440	0.912839	0.034722	7.750300	1.1220
ScHK2	25	*λ^U^*	5.385780	21	0.045398	0.923280	0.031322	10.90060	1.0240
ScHK2	36	*λ^U^*	7.248180	18	0.040294	0.930381	0.029326	13.90760	0.9188
ScHK2	49	*λ^U^*	9.160180	15	0.036641	0.935141	0.028218	16.66100	0.8189
ScHK2	64	*λ^U^*	11.06320	17	0.033702	0.938735	0.027563	19.12850	0.7290
HsHK2	1	*λ^E^*	8.649300	8	0.091012	0.908980	0.000008	13.12310	0.5172
HsHK2	4	*λ^E^*	21.54300	12	0.070280	0.929719	0.000001	25.56560	0.1867
HsHK2	9	*λ^E^*	28.92340	16	0.061383	0.938615	0.000002	31.59840	0.0925
HsHK2	16	*λ^E^*	32.96630	20	0.056368	0.943629	0.000003	34.76250	0.0545
HsHK2	25	*λ^E^*	35.34790	25	0.053100	0.946886	0.000015	36.61110	0.0357
HsHK2	36	*λ^E^*	36.85580	26	0.050721	0.949259	0.000019	37.78430	0.0252
HsHK2	49	*λ^E^*	37.86820	22	0.048855	0.951144	0.000001	38.57600	0.0187
HsHK2	64	*λ^E^*	38.58160	26	0.047409	0.952590	0.000002	39.13760	0.0144

**Table 6:** Optimal parameters λ* for the SE-scenario with ScHK2 (top) and HsHK2 (bottom), for one bead and different runtimes *T* on a mesh with *n_h_* = 820.

Enzyme	*T*	****λ****^(0)^	*J*^0^	k*	$\lambda {\;}_{\text{inv}}^{*}$	$\lambda {\;}_{\text{hk}}^{*}$	$\lambda {\;}_{\text{pgi}}^{*}$	J*	$\frac{J^{*}-J^{\text{0}}}{J^{\text{0}}}$
ScHK2	1800	*λ^U^*	0.053486	17	0.138824	0.808964	0.052212	0.100827	0.8851
ScHK2	3600	*λ^U^*	0.228066	16	0.120404	0.815447	0.064149	0.446978	0.9599
ScHK2	5400	*λ^U^*	0.524945	12	0.109005	0.826746	0.064249	1.060920	1.0210
ScHK2	7200	*λ^U^*	0.941239	11	0.100778	0.837288	0.061934	1.944520	1.0659
ScHK2	9000	*λ^U^*	1.473430	13	0.094578	0.845965	0.059457	3.092240	1.0987
ScHK2	10,800	*λ^U^*	2.117810	19	0.089829	0.852873	0.057298	4.495210	1.1226
ScHK2	12,600	*λ^U^*	2.870590	19	0.085547	0.859325	0.055128	6.142920	1.1400
ScHK2	14,400	*λ^U^*	3.727980	16	0.082116	0.864621	0.053264	8.024060	1.1524
HsHK2	1800	*λ^E^*	2.246900	12	0.104737	0.895244	0.000019	4.001410	0.7809
HsHK2	3600	*λ^E^*	8.649300	8	0.091012	0.908980	0.000008	13.12310	0.5172
HsHK2	5400	*λ^E^*	17.80560	8	0.083651	0.916269	0.000080	24.82340	0.3941
HsHK2	7200	*λ^E^*	28.77910	9	0.078851	0.921146	0.000003	38.03850	0.3217
HsHK2	9000	*λ^E^*	31.53360	10	0.075383	0.924615	0.000002	52.22680	0.6562
HsHK2	10,800	*λ^E^*	54.15470	9	0.072366	0.927633	0.000001	67.09490	0.2389
HsHK2	12,600	*λ^E^*	67.98330	9	0.070322	0.929672	0.000006	82.46000	0.2129
HsHK2	14,400	*λ^E^*	82.34300	9	0.068190	0.931809	0.000001	98.20600	0.1926

In the presence of the hexokinase HsHK2, we find from the first arrangement that the optimal stoichiometry for a runtime of 1 h is between 5 and 9% for invertase, between 91 and 95% for hexokinase and approximately 0% for the phosphoglucose isomerase. With an increasing number of beads, the proportion of hexokinase increases and the proportion for invertase decreases. The exact results are listed in Table 5. From the arrangement (O.2), we find that the optimal stoichiometry for one bead is between 7 and 10% for invertase and between 90 and 93% for the hexokinase again with an increase in the proportion of binding sites loaded with HsHK2 in expense of the proportions for ScINV. As in arrangement (O.1), the optimal proportion of phosphoglucose isomerase is approximately 0%. The exact results are again listed in Table 6. We stress that the optimization results in Table 5 correspond to local maxima.

## 4 Discussion

In this paper, we developed mathematical techniques for the simulation and optimization of metabolic processes in biological microreactors with membrane-bounded subcompartments. The results from our models will be incorporated into the design of a microreactor and will be validated by experiments with this microreactor. The microreactor is based on a microfluidic system, and consists of chambers separated by membranes which carry specific transporters for input of substrates and export of products. Inside the chambers, magnetic nano-beads carrying multienzyme-complexes are distributed, and immobilized by an outer magnetic field.

We have set up a mathematical model describing the spatio-temporal dynamics of the metabolite concentrations involved in the assembled metabolic pathway, and develop efficient and accurate approaches for the numerical simulation of this model. Furthermore, we show that model-based optimization for such systems is feasible by the methods presented. These approaches are applied to the proof-of-concept microreactor carrying out the synthetic pathway for the conversion of sucrose to glucose-6-phosphate. The obtained results shed light on the following important questions linked to the design and functionality of microreactors.

### 4.1 Are Spatially Resolved Models Necessary in the Description of Biochemical Microreactors?

The simulation of the distribution of metabolites, e.g., that of G6P in the presence of the enzyme HsHK2 (Figure 4), shows spatial patterns, which differ for different experimental arrangements. In case of completely loaded beads (Figure 4, left) the highest concentration of G6P can be found around the beads. If the beads are just partially loaded (Figure 4, right), a concentration gradient across the chamber prevails. In this case, the total number of enzymes distributed to the beads corresponds to the number of enzymes on one completely loaded bead. These spatial patterns in the metabolite distributions confirm the demand of spatially resolved models in the description of metabolic processes carried out in synthetic microreactors and, ultimately in living cells, which are more complex in architecture. Furthermore, the limiting effect of diffusion also shows that spatial effects have to be taken into account. This is visible in arrangement (S.2), where in the initial phase of the simulation the production rate of G6P at the beads decreases with increasing number of beads (Figure 6, upper figures); however, the outflow rate of G6P is higher for a large number of beads. This results from the fact that for high number of beads there are more beads close to the outflow boundary Γ*_e_*, and consequently G6P diffusion to the boundary Γ*_e_* needs less time. We mention, however, that for longer runtimes, the higher production rate of G6P for lower numbers of beads is predominating, and the outflow rates of G6P are higher for less number of beads (Figure 6, lower pictures).

### 4.2 Has Spatial Organization and Microcompartmentation of Enzymes the Potential to Influence the Efficiency of Metabolic Pathways?

Comparing the outflow rate of the product G6P for a fixed total enzyme quantity which is distributed on different numbers of beads, it turns out that the production rate can be increased with decreasing number of beads (Table 4; Figure 6, lower figures), especially, assembling all enzymes on one bead leads to the maximal outflow of G6P. This remains true also after comparing the upper scenarios with the scenario, in which enzymes are distributed in the bulk fluid within the reactor chamber. This last scenario turns out to lead to the lowest G6P outflow. For the scenario with one bead and the hexokinase HsHK2, the outflow of G6P is approximately 60% higher than the total outflow for the scenario with enzymes distributed within the bulk fluid with the same hexokinase (Table 4). These results suggest that microcompartmentation of enzymes increases the total output of G6P, not only in the model but also *in vitro*.

### 4.3 What Are the Limitations for the Productivity of the Modeled (Synthetic) Membrane-Bounded Microreactors?

The simulation results for the arrangement (S.1) (with completely loaded beads) show that the outflow rates for G6P reach saturation, in spite of the fact that the activity inside the chamber increases with increasing number of beads. This can directly be seen for the hexokinase HsHK2 (Figure 5, right bottom), whereas for ScHK2 this effect does not occur up to the run time of 3 h (Figure 5, left bottom). However, our computations show that at later time, this limiting effect also occurs for this hexokinase. This suggests that the limiting factor of the G6P outflow is the activity of the transporters in the membrane Γ*_e_*. Finally, we mention that for the arrangement (S.2) the outflow rates are not reaching saturation, which implies that the activity of the transporters is not limiting the outflow for the chosen enzyme concentration [*E*]_full_.

### 4.4 Can Optimal Design Help to Improve the Yield of a Biosynthetic Pathway?

**Figure 7:**
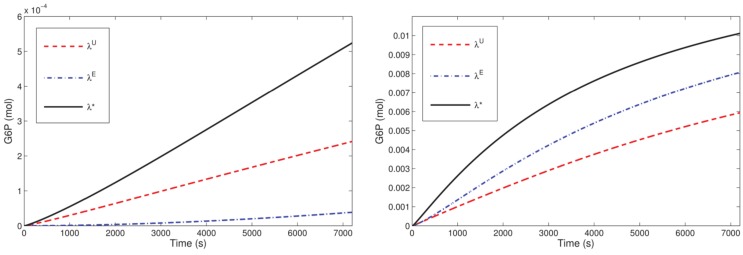
G6P outflow for the SE-scenario with *n_b_* = 1 compared for the uniform (*λ^U^*), equal activity (*λ^E^*) and optimal (λ*) stoichiometry for *T* = 7200 and ScHK2 (left) and HsHK2 (right) computed with Δ*t* = 0.25 and *n_h_* = 820.

**Figure 8:**
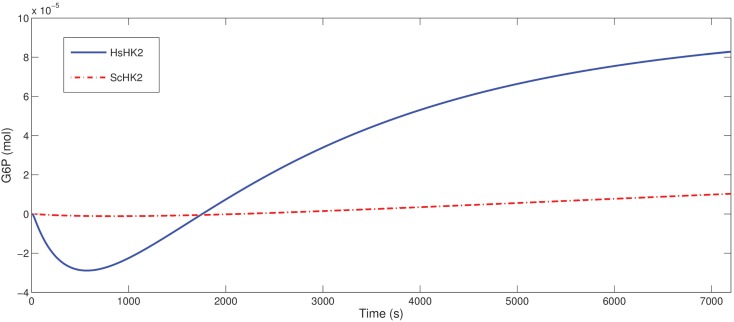
Predicted difference of in the G6P outflow for the SE-scenario with *n_b_* = 1 up to *T* = 7200 s comparing the optimal stoichiometry λ* obtained for *T* = 7200 and that for *T* = 1800 both for ScHK2 (dash-dotted line) and HsHK2 (solid line) from Table 6.

Our optimization results for the microreactor in the SE-scenario with the hexokinase ScHK2, show that after a run time *T* = 3600 s, the total outflow of G6P, denoted by J*, for the optimal stoichiometry λ* is on average twice as much as the total outflow *J*^0^ for the uniform stoichiometry *λ^U^*, and even much more than for the stoichiometry *λ^E^*, e.g., 2746% in case of one bead. For one bead, this improvement is also visualized in Figure 7 (left). When run time is successively increased (for a reactor with one bead) starting from *T* = 1800 s, again the optimal stoichiometries enhance the total outflow of the desired product G6P on average twofold compared to the uniform stoichiometry and even much more compared to the stoichiometry *λ^E^*. The comparison of the G6P outflow over time for stoichiometries computed for different runtimes shows that a larger proportion of binding sites loaded with ScINV leads to a higher G6P outflow at the initial phase while a larger proportion of binding sites loaded with ScHK2 leads to a higher G6P outflow on the long run (Figure 8).

Optimization of the microreactor setup with the hexokinase HsHK2 suggests that the phosphoglucose isomerase can be eliminated for the run times considered in the optimization scenarios. Furthermore, in contrast to the results for ScHK2, the degree of improvement of the optimal stoichiometries is between 50% (for one bead) and 1% (for 64 beads) compared to the stoichiometry computed from equal activity and slightly higher compared to the uniform stoichiometry. For one bead, the improvement is also visualized in Figure 7 (right). When time is successively increased (for a reactor with one bead) from *T* = 1800–14,400 s, the optimal stoichiometries now enhance the total outflow of G6P. This enhancement is 20% up to 78% compared to the stoichiometry computed from equal activity and slightly more compared to the uniform stoichiometry, e.g., 147% for *T* = 1800. The observation obtained for ScHK2 concerning the role of the invertase and hexokinase for the initial phase and on the long run is now even more pronounced (Figure 8).

**Figure 9:**
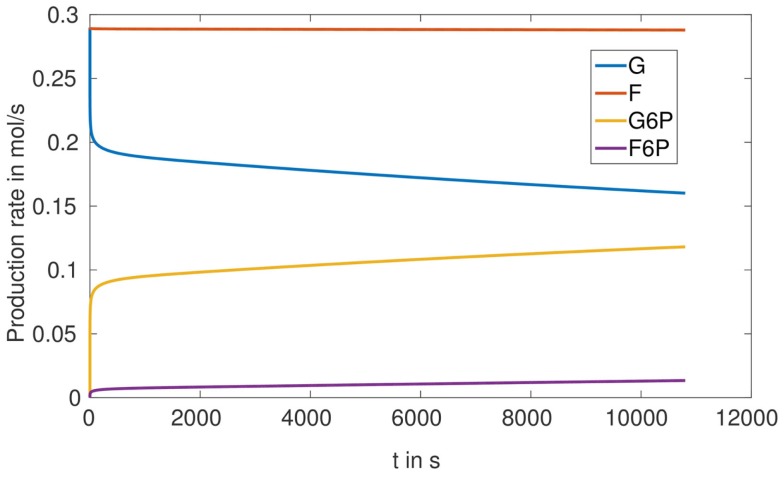
**Production rates in $\frac{\text{mol}}{s}$ for one fully loaded bead in the SE-scenario with the hexokinase HsHK2**.

To understand the optimization result obtained for the proportion of phosphoglucose isomerase (the enzyme catalyzing the reaction G6P ⇌ F6P), in case of the microreactor with HsHK2, namely that this enzyme should be better eliminated, we simulated the production rates for all metabolites, for one completely loaded bead (Figure 9). We see that, with respect to our considered time interval, the production rates for G and F increase very fast to a maximum-level around 0.3 $\frac{\mathrm{mol}}{s}$. The reaction rates in Table 2 imply that the conversion of S into F is constant, since the concentration of S is constant. Furthermore, Figure 9 shows that the production rate of F6P increases in time, but the production rate of F remains nearly constant. Hence, we conclude that there is a metabolic flux from G6P to F6P, what contradicts the reactor to obtain a maximal outflow of G6P, and should be avoided by considering a small activity of phosphoglucose isomerase.

We stress that none of the reported maxima can be guaranteed to be global optima. Nevertheless, concerning the quality of the maxima, we note that all search paths from *λ*^(0)^ to λ* transverse a large part of the parameter space and are associated with a significant decrease in the costs. Moreover, we have tested various initial points *λ*^(0)^ for the scenario with one bead, HsHK2 and *T* = 3600. In all cases, we have observed convergence to the same λ*. We conclude that the reported local maxima are reasonable candidates for an optimized stoichometry for the respective microreactor.

Finally, we emphasize that the simulation and optimization approaches developed in this work can be repeated with minor adaptions for more complex biochemical microreactors and for other optimization parameters, like, e.g., other real parameters entering the reaction kinetics.

## Author Contributions

MN-R and LV gave substantial contributions to the Abstract and Introduction. MG and MN-R gave substantial contributions to the conception of the Derivation of the Mathematical Model, see Section 2.1. TE and PK gave substantial contributions to the conception of the Numerical Analysis and Simulations, see Section 2.2 and 3.1. FH and GL gave substantial contributions to the conception of the Optimization Methods and Results, see Section 2.3 and 3.2. LV gave substantial contributions to the acquisition, analysis, and interpretation of data for the work. All authors contributed essentially in the discussion of the results, see Section 4. Also, all authors have revisited the work critically for important intellectual content, approved the version to be published, and agreed to be accountable for all aspects of the work.

## Conflict of Interest Statement

The authors declare that the research was conducted in the absence of any commercial or financial relationships that could be construed as a potential conflict of interest.

## Footnotes

^1^Gahn, M., Neuss-Radu, M., and Knabner, P. (2015). Homogenization of reactiondiffusion processes in a two-component porous medium with nonlinear flux conditions at the interface. *SIAM J. Appl. Math*. (Under Review).
