# Supplementary material for: Model-Based Design of Biochemical Microreactors
Source: Front Bioeng Biotechnol. 2016 Feb 15;4:13. doi: 10.3389/fbioe.2016.00013 (PMC4753381; doi:10.3389/fbioe.2016.00013)
Supplement: Supplementary file 1 [file Supplementary_Material.PDF]

Supplementary Material: Model based design of biochemical micro-reactors

Tobias Elbinger [1], Markus Gahn [1], Maria Neuss-Radu [1,*], Falk M. Hante [2], Lars M. Voll [3], Günter Leugering [2] and Peter Knabner [1]

[1] *Chair of Applied Mathematics 1, Mathematics Department, University of Erlangen-Nuremberg, Erlangen, Germany*
[2] *Chair of Applied Mathematics 2, Mathematics Department, University of Erlangen-Nuremberg, Erlangen, Germany*
[3] *Chair of Biochemistry, Biology Department, University of Erlangen-Nuremberg, Erlangen, Germany*

Correspondence*:
Maria Neuss-Radu
Mathematics Department, University of Erlangen-Nuremberg, Cauerstr. 11, Erlangen, 91058, Germany, maria.neuss-radu@math.fau.de

Engineering synthetic metabolons: from metabolic modelling to rational design of biosynthetic devices

1 SUPPLEMENTARY TABLES AND FIGURES

Detailed information concerning the number of points, triangles and edges of the meshes used for spatial discretization can be found in Table 1 for $\Omega_c = (0, 3000\mu m)^2$ and in Table 2 for $\Omega_c = (0, 500\mu m)^2$. Exemplary meshes are shown in Figures 1 – 3 for the larger domain and in Figures 4 – 6 for the smaller domain. The meshes were created using the software gmsh.

Supplementary Table 1. Number of points, triangles and edges of the used meshes for $\Omega_c = (0, 3000\mu m)^2$ and $n_b \in \{0^2, 1^2, ..., 6^2, 12^2, ...60^2\}$.

n_b	Points	Triangles	Edges
0	7766	15226	22991
1	7751	15190	22941
4	7760	15190	22953
9	7963	15566	23537
16	7863	15324	23202
25	8440	16424	24888
36	8042	15562	23639
144	9861	18552	28556
324	12585	22920	35828
576	18606	33450	52631
900	22629	39552	63080
1296	28598	49114	79007
1764	33685	56480	91928
2304	43889	73648	119840
2916	55493	93184	151592
3600	54433	86960	144992

Supplementary Table 2. Number of points, triangles and edges of the used meshes for $\Omega_c = (0, 500\mu m)^2$ and $n_b \in \{1^2, 2^2, ..., 8^2\}$.

n_b	Points	Triangles	Edges
1	450	820	1270
4	507	916	1426
9	598	1068	1674
16	647	1124	1786
25	845	1466	2335
36	1053	1816	2904
49	1172	1976	3196
64	1426	2394	3883

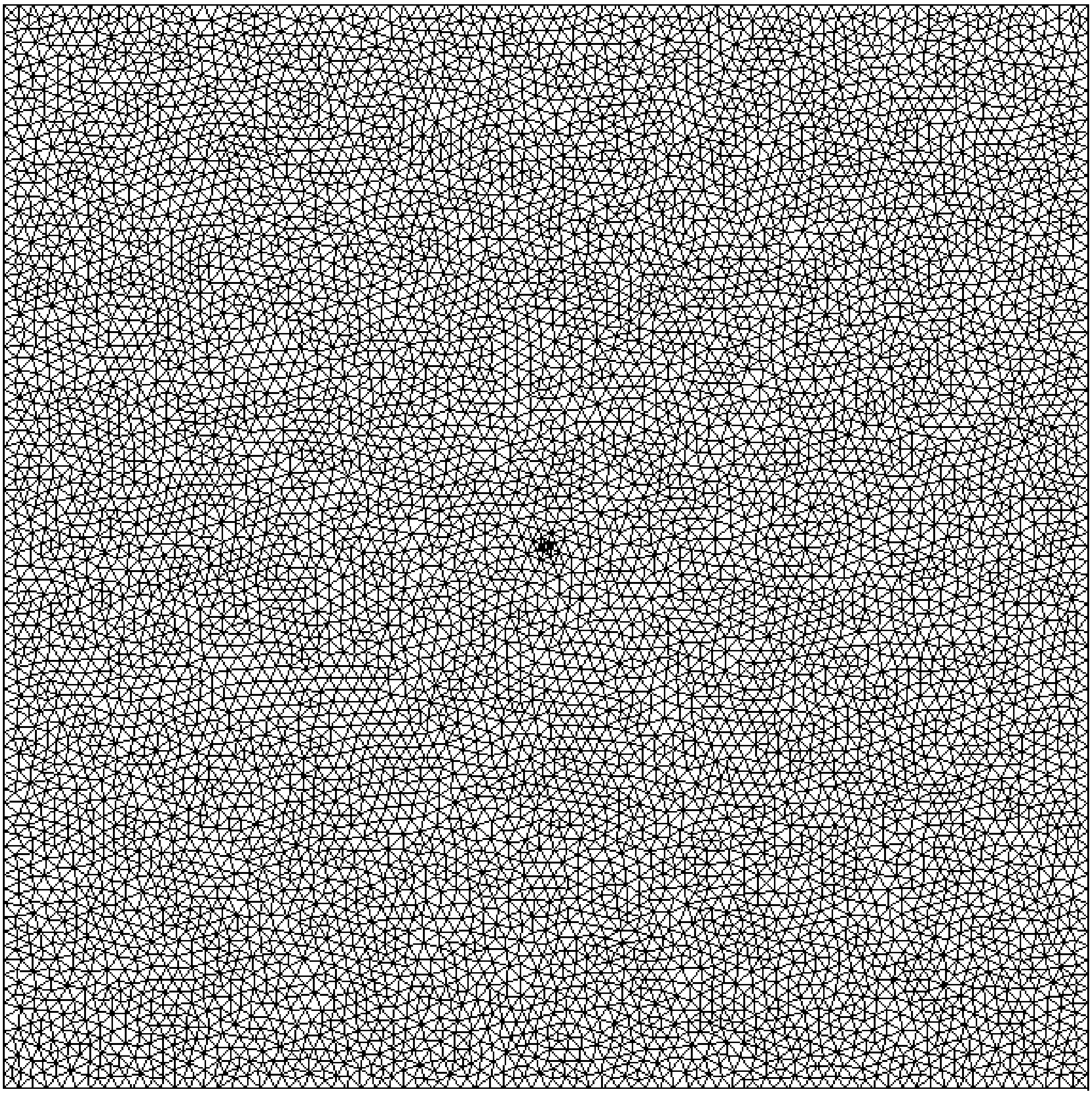

Supplementary Figure 1. Mesh for $\Omega_c = (0, 300\mu m)^2$ and $n_b = 1$.

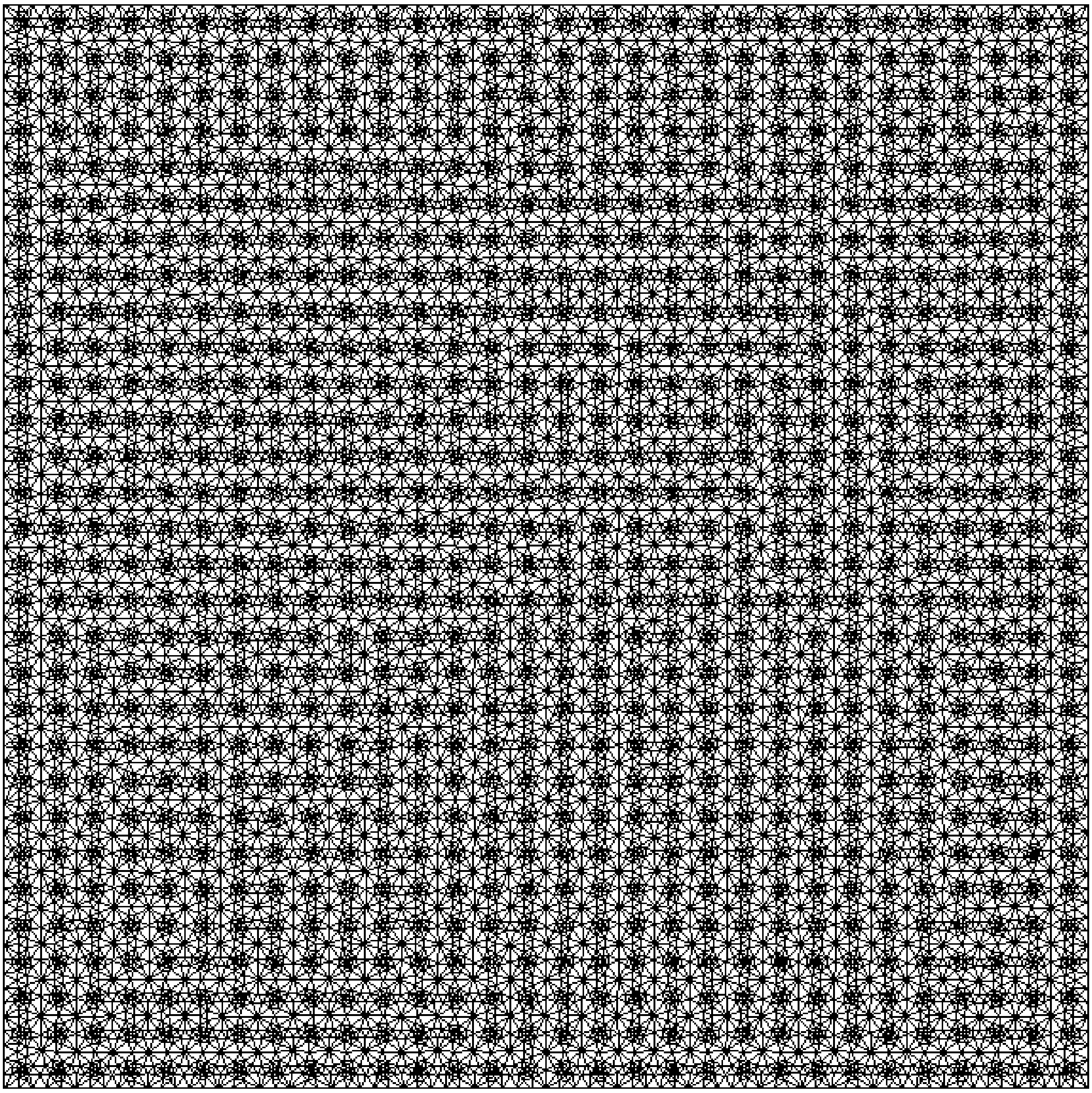

Supplementary Figure 2. Mesh for $\Omega_c = (0, 300\mu m)^2$ and $n_b = 30$.

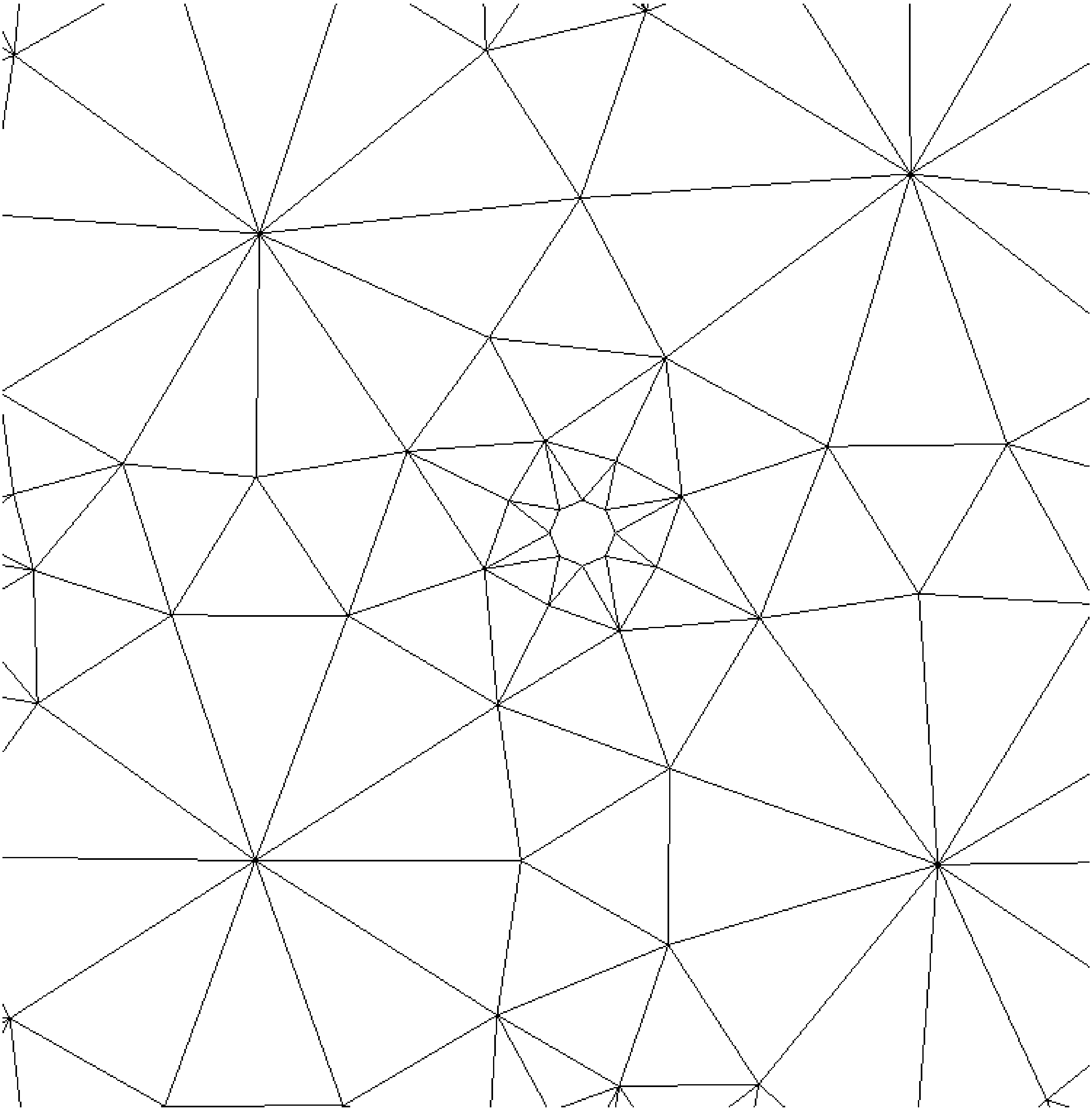

Supplementary Figure 3. Mesh for $\Omega_c = (0, 300\mu m)^2$ and $n_b = 30$, zoom on a bead.

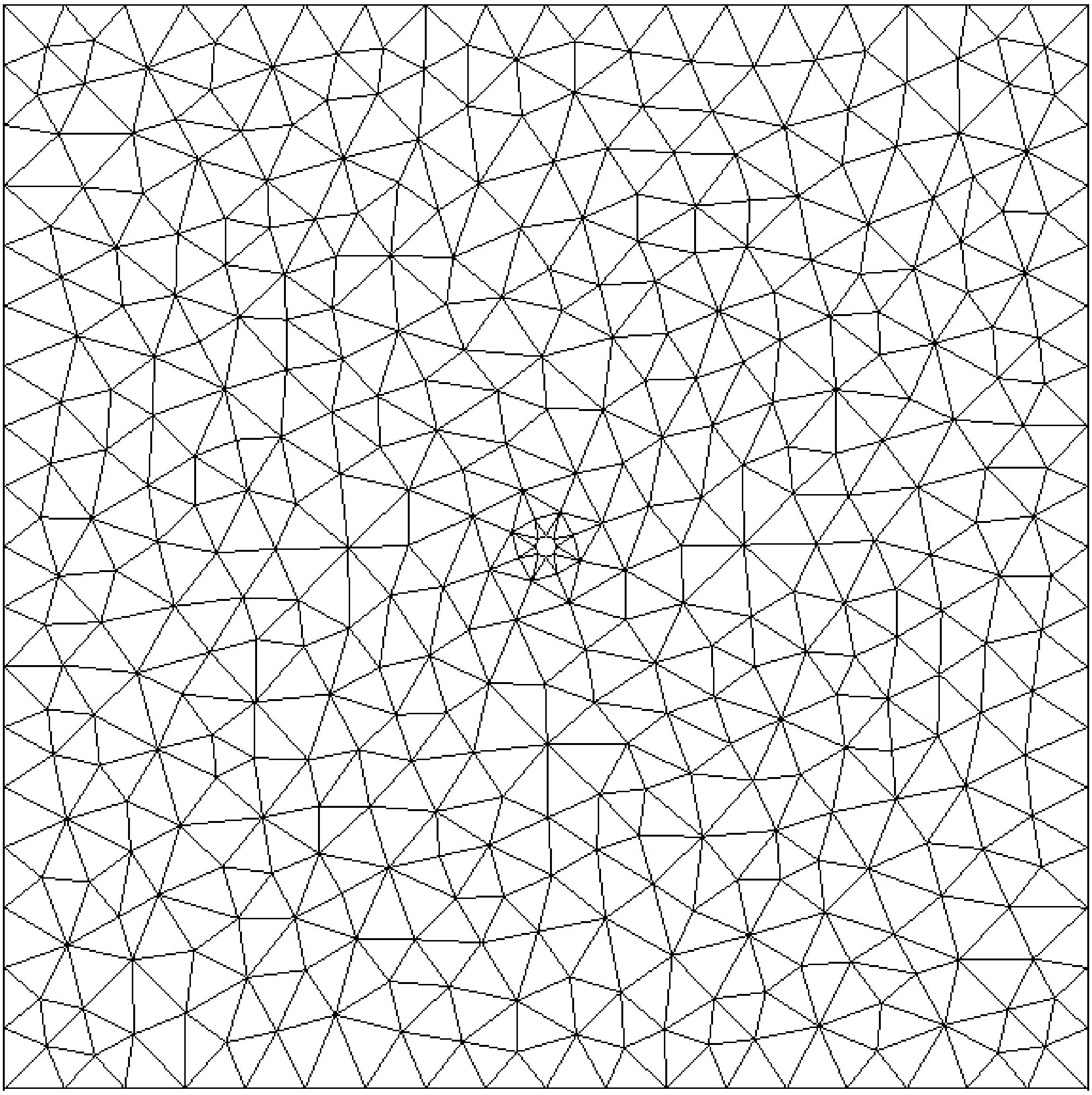

Supplementary Figure 4. Mesh for $\Omega_c = (0, 50\mu m)^2$ and $n_b = 1$.

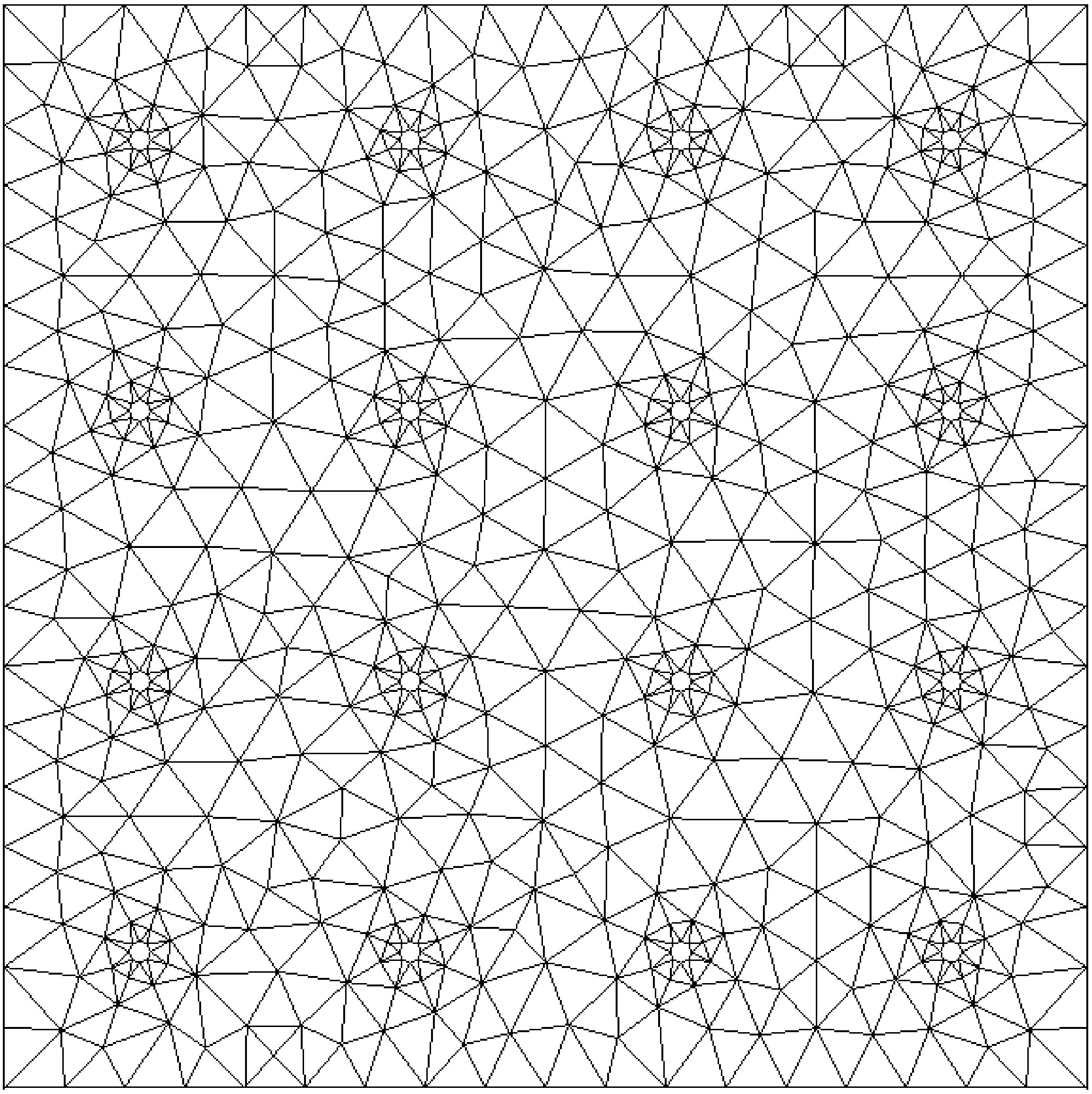

Supplementary Figure 5. Mesh for $\Omega_c = (0, 50\mu m)^2$ and $n_b = 16$.

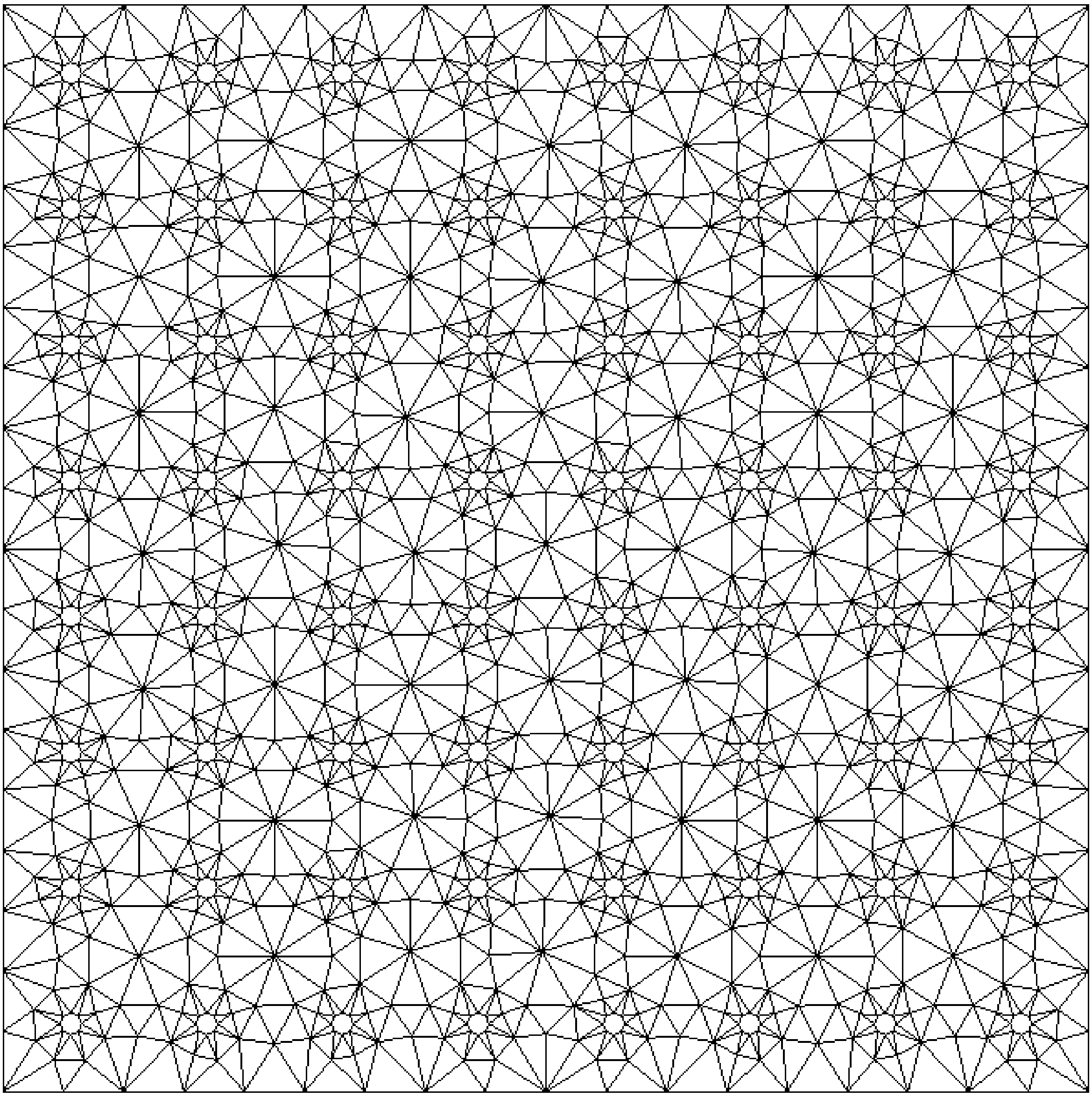

Supplementary Figure 6. Mesh for $\Omega_c = (0, 50\mu m)^2$ and $n_b = 64$.
